# Study on Efficient Adsorption Mechanism of Pb^2+^ by Magnetic Coconut Biochar

**DOI:** 10.3390/ijms232214053

**Published:** 2022-11-14

**Authors:** Yonghua Xu, Youpei Qu, Yujia Yang, Bin Qu, Rui Shan, Haoran Yuan, Yong Sun

**Affiliations:** 1 College of Electrical and Information, Northeast Agriculture University, Harbin 150030, China; 2 College of Engineering, Northeast Agriculture University, Harbin 150030, China; 3 Key Laboratory of Agricultural Renewable Resources Utilization Technology and Equipment in Cold Areas of Heilongjiang Province, Harbin 150030, China; 4 College of Arts and Sciences, Northeast Agriculture University, Harbin 150030, China; 5 Guangzhou Institute of Energy Conversion, Chinese Academy of Sciences, Guangzhou 510640, China

**Keywords:** coconut bark, biochar, adsorption mechanism, Pb^2+^

## Abstract

Lead ion (Pb^2+^) in wastewater cannot be biodegraded and destroyed. It can easily be enriched in living organisms, which causes serious harm to the environment and human health. Among the existing treatment technologies, adsorption is a green and efficient way to treat heavy metal contamination. Novel KMnO_4_-treated magnetic biochar (KFBC) was successfully synthesized by the addition of Fe(NO_3_)_3_ and KMnO_4_ treatment during carbonization following Pb^2+^ adsorption. SEM-EDS, XPS, and ICP-OES were used to evaluate the KFBC and magnetic biochar (FBC) on the surface morphology, surface chemistry characteristics, surface functional groups, and Pb^2+^ adsorption behavior. The effects of pH on the Pb^2+^ solution, initial concentration of Pb^2+^, adsorption time, and influencing ions on the adsorption amount of Pb^2+^ were examined, and the adsorption mechanisms of FBC and KFBC on Pb^2+^ were investigated. The results showed that pH had a strong influence on the adsorption of KFBC and the optimum adsorption pH was 5. The saturation adsorption capacity fitted by the model was 170.668 mg/g. The successful loading of manganese oxides and the enhanced oxygen functional groups, as evidenced by XPS and FTIR data, improved KFBC for heavy metal adsorption. Mineral precipitation, functional group complexation, and π-electron interactions were the primary adsorption processes.

## 1. Introduction

Water contamination, particularly heavy metal pollution, is a huge global concern that poses irreparable harm to human health [1]. Pb^2+^ is one of the most toxic metals in nature. Lead has been widely employed in recent years in manufacturing and residential applications such as synthetic paint mining and metal smelting and processing [2]. The central nervous system can be damaged by excessive amounts of lead in the environment; children living in these environments for long periods with low levels of lead may also suffer from reduced intelligence [3]. Therefore, the treatment of lead-containing wastewater requires an environment-friendly and economical treatment technology. Researchers have proposed several methods to remove heavy metal ions, including but not limited to chemical precipitation, ion exchange, adsorption, and electrochemical methods [4]. Compared to other methods, biochar adsorbents have a distinct advantage in wastewater treatment due to their low cost, good applicability, and selectivity [5].

Coconut peel is a typical abundant yield and cheap agricultural waste mainly used to produce low-value-added products. The preparation of biochar by coconut peel to treat heavy metals is an effective means of high-value utilization, so as to achieve waste treatment. Conventional pyrolysis of biomass is widely used in the preparation of biochar. In an oxygen-limited environment filled with inert gases, the lignocellulose and fats in the feedstock are broken down at high temperatures, reducing the hydrogen and oxygen content and increasing the carbon content [6]. Biomass with low water content (less than 30%) is usually used for pyrolysis. Coconut shell biochar has great potential to adsorb Pb^2+^ compared to traditional adsorbents and activated carbon and has become a viable option to address Pb^2+^ pollution. The specific surface area of coconut shell biochar is much lower than that of commercial activated carbon; but coconut shell biochar can remove heavy metal ions from water more effectively than activated carbon due to the presence of a large number of functional groups on the surface of the biochar [7]. Metal oxides-loaded biochar such as MgO, MnOx, and CeO_2_ have a higher adsorption capacity for Pb^2+^ [8]. Magnetic biochars loaded with iron-based composite oxides also sorbed up to 61.25 mg/g [9]. In recent years, researchers have often loaded metal oxides on the surface of the biochar to expand their adsorption capacity [10,11]. Magnetic biochar is easy to separate and recover, and a load of iron oxide can realize the magnetic modification of the biochar. Bimetal oxide prepared at a high temperature has good stability and metal ions are not easily released, which can effectively reduce the risk of secondary pollution. In this study, we made the biochar magnetic and attached another metal oxide in order to obtain adsorbents with high adsorption capacity and easy separation. Since there are many factors affecting the adsorption of heavy metals by the biochar and the mechanism of action is complex, the main adsorption mechanisms can vary even for a given heavy metal. The adsorption mechanisms of the biochar are van der Waals forces, precipitation, electrostatic attraction, ion exchange, and π-bond complexation [12]. In studying the mechanism of Pb^2+^ adsorption by the biochar, Wang found that its physicochemical properties are the main factor affecting the adsorption performance, which is jointly determined by the raw material and pyrolysis conditions of the biochar [13]. Lu’s study found that the adsorption of lead by sludge biochar mainly involves coordination with organic hydroxyl and carboxyl functional groups, as well as co-precipitation or complexation on mineral surfaces [14]. The adsorption of heavy metals by biochar is not a single mechanism but usually a combination of multiple reaction mechanisms. Therefore, it is necessary to study the adsorption mechanism of Pb^2+^ by coconut shell biochar and develop a modified biochar process for the removal of Pb^2+^ from wastewater using renewable biomass as a raw material.

In this paper, KMnO_4_-modified magnetic biochar was prepared by coconut peel through impregnation pyrolysis to strengthen the adsorption capacity of magnetic biochar for Pb^2+^; the manganese oxide was stably attached to the biochar after high-temperature calcination. Batch adsorption experiments were conducted using biochar to investigate the adsorption kinetics, adsorption isotherms, the effect of alkali metal ion coexistence, the quantification of adsorption mechanism, and the effect of pH on adsorption. Finally, XPS, XRD, and FTIR of the adsorbed biochar were characterized to compare the changes of chemical bonding, crystal structure, and surface functional groups of the biochar for qualitative analysis of the adsorption mechanism; the adsorption mechanism was quantitatively analyzed by dividing the adsorption into functional group-dominated, mineral-dominated, and π-electron-dominated parts using the acid washing method. Through this study, a new type of coconut shell biochar was developed to provide a new idea for the industrial treatment of Pb^2+^ wastewater.

## 2. Results and Discussion

### 2.1. Characterization of Biochar

**Figure 1:**
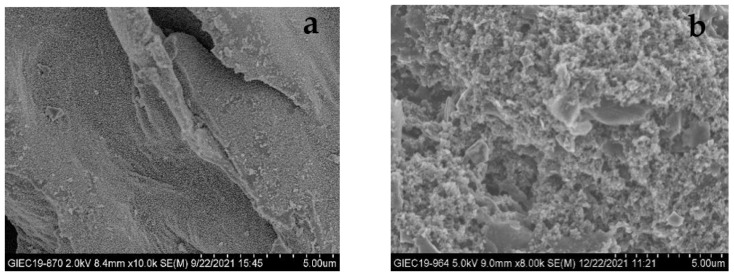
SEM images of (**a**) FBC and (**b**) KFBC.

FBC has a lamellar structure with no obvious pore structure observed on the surface (Figure 1), but its surface is uniformly loaded with iron oxide nanoparticles and the surface is relatively flat with some folds. The collapsed structure of the KFBC surface may be due to the strong oxidizing properties of potassium permanganate and the metal oxides loaded on the surface of the biochar.

**Figure 2:**
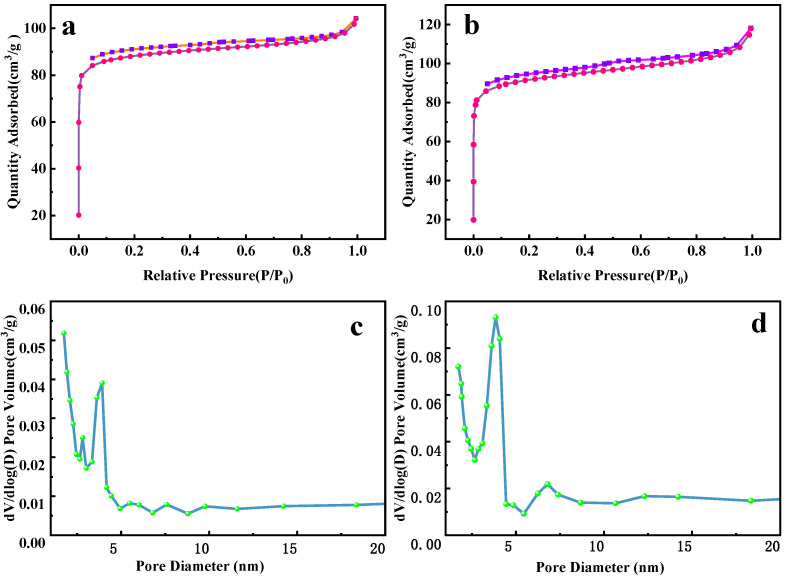
N_2_ adsorption and desorption curves for (**a**) FBC, (**b**) KFBC, and pore size distribution for (**c**) FBC, (**d**) KFBC.

The adsorption–desorption curves of FBC and KFBC are not closed (Figure 2). This may be due to the complex pore structure of the biochar, which is prone to flexible pores or ink bottle shaped pores. The pore diameter shrinks after gas adsorption, making it difficult for the adsorbed gas to desorb, resulting in the curves not being closed. For FBC, the peak of the pore size distribution was at 1.74 nm, and, for KFBC, the peak was at 3.84 nm, indicating that the modified magnetic biochar had a mesoporous structure with a slight increase in KFBC pore size, probably due to the strong oxidation of KMnO_4_. The pores of the biochar are corrosive. Compared to FBC, the pore volume expanded from 0.030 cm^3^/g to 0.053 cm^3^/g, as shown in Table 1. The increase in pore volume resulted in more active sites for biochar adsorption. 

**Figure 3:**
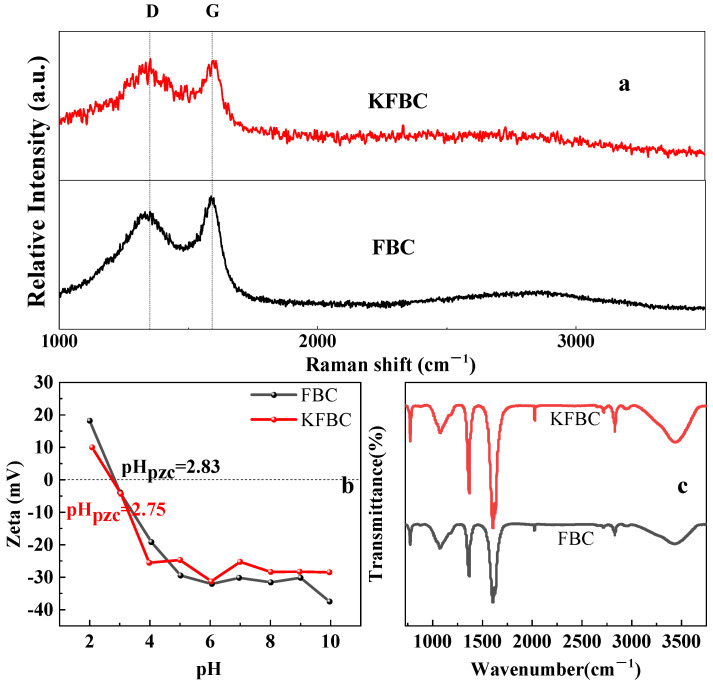
Raman spectra of FBC and KFBC (**a**), biochar zeta potential at different pH values (**b**), FTIR spectra of FBC and KFBC (**c**).

Peak D represents the vibration of sp3 hybridization, manifested by the stacking of the graphite structure of the carbon material and the defects produced by the edge state; G peak represents the expansion vibration of sp2 hybridization, which shows the degree of graphitization of the biochar, namely, the ideal graphite lattice (Figure 3a). The band of the G peak is located at 1389–1398 cm^−1^ and the band of the D peak is located at 1340–1357 cm^−1^. The intensity ratio of the D peak to the G peak can assess the degree of defects on the surface of the biochar. The value of I_D_/I_G_ of the biochar increased from 0.929 to 1.334 after modification; the KMnO_4_ modification led to an increase in the disorder and defect degree of the biochar. It was shown that the modification of KMnO_4_ promoted the production of more oxygen-containing functional groups, thus enhancing the adsorption capacity of the adsorbent for Pb^2+^. When the solution pH (2.83, 2.75) was less than the zero point charge, the surface of the material was positively charged and the electrostatic adsorption of Pb^2+^ was weak. As the pH increased, the acidic functional groups on the surface of the biochar were deprotonated. When the solution pH was greater than the zero point charge, the surface of the biochar was negatively charged and the electrostatic attraction was enhanced. As the solution pH increased, the zeta potential value decreased significantly. The stronger the alkaline conditions, the greater was the degree of deprotonation of the oxygen-containing functional groups and the negative charge on the biochar increased with increasing pH. As shown in Figure 3b, the pH_pzc_ of FBC and KFBC was 2.83 and 2.75, respectively. It was, thus, clear that when pH > 5, the biochar was in a negative state, which was more favorable for the adsorption of cations. Compared with the unmodified biochar, the modified biochar increased the positive charge and decreased the negative charge on the surface of the biomass due to the loading of more metal oxides, resulting in a slight increase in the zeta potential value of the modified biochar [15].

Functional groups on the surface of FBC and KFBC were determined by FTIR (Figure 3c). Compared to FBC, the modified biochar showed similar peak patterns and no new peaks at the same range of wave numbers, indicating that there was no increase in the type of functional group. The absorption peaks at 3420 cm^−1^ and 1627 cm^−1^ were attributed to the stretching vibrations of hydroxyl (-OH) and carbonyl (C=O), respectively [16]. The peak at 2922 cm^−1^ was attributed to the extension of methyl and methylene (C-H) [17]. At 1606 cm^−1^ was the absorption peak of aromatic C=C. The peak intensity of the absorption peak of the modified biochar became stronger, indicating an increase in oxygen-containing functional groups.

**Figure 4:**
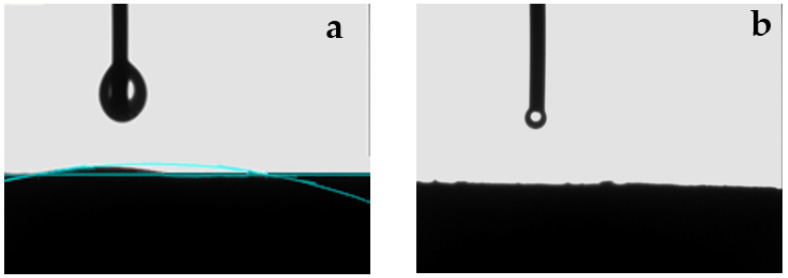
Images of contact angle of (**a**) FBC and (**b**) KFBC with deionized water.

The wettability of the biochar is also critical for adsorption. The increased hydrophilicity of the modified biochar is due to the production of more oxygen-containing functional groups (such as hydroxyl groups) on its surface; the oxygenated functional group has strong adsorption between water molecules, and the hydrophilic property of the biochar is directly related to the content of oxygenated functional group. The water contact angle decreases as the concentration of the functional groups increases [18,19]. The contact angle of the FBC before modification was 10° and the contact angle of the KFBC after modification was 0° (Figure 4), indicating that the modification enhanced the hydrophilic properties of the biochar, which facilitated the transfer of pollutants to the adsorbent surface, promoted the transport of substances, and enhanced the adsorption capacity. 

**Figure 5:**
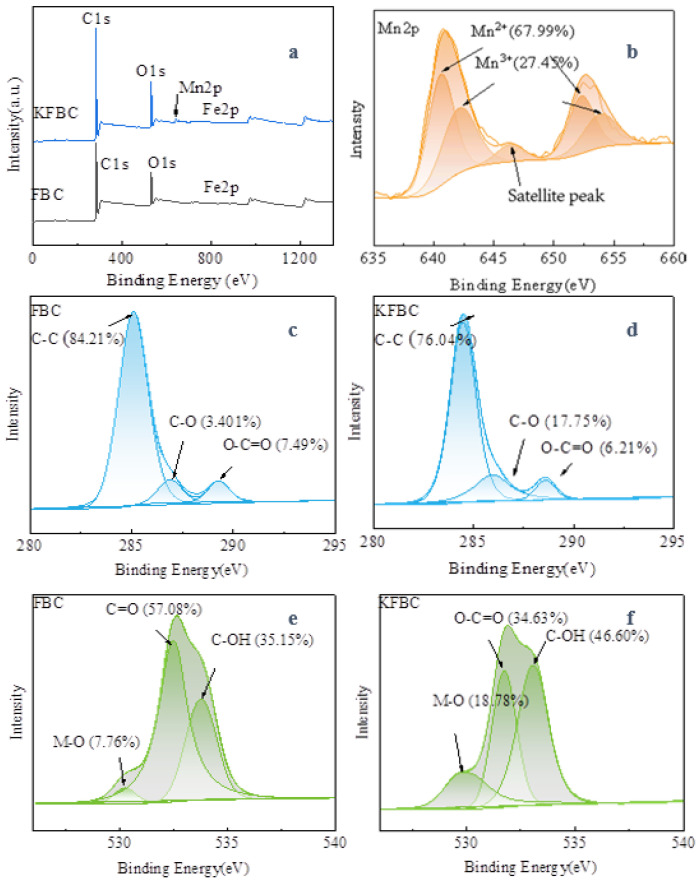
XPS spectra of wide scan of FBC and KFBC (**a**), Mn2p of KFBC (**b**), C1s of FBC(**c**), C1s of KFBC (**d**), O1s of FBC (**e**), and C1s of KFBC (**f**).

**Figure 6:**
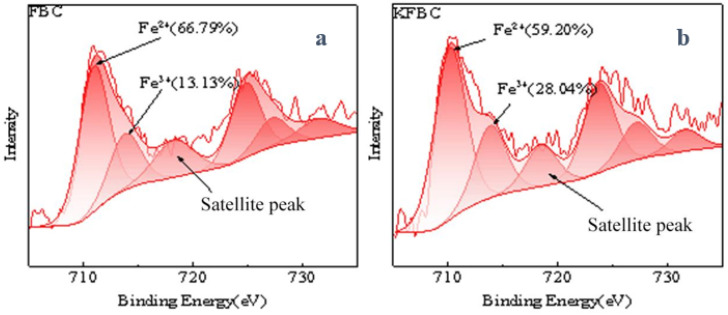
XPS spectra of Fe2p of FBC (**a**) and Fe2p of KFBC (**b**).

XPS enables the semi-quantitative determination of the surface composition and the chemical valence state of the biochar. The O1s peak of the biochar with a binary metal oxide load was higher than the biochar with a binary metal oxide load (Figure 5a), indicating that the surface oxygen content of the modified biochar was increased. The Mn2p spectra contained two asymmetric peaks at 641.1 eV and 652.6 eV, representing Mn2p3/2 and Mn2p1/2, respectively, and a satellite peak of Mn^2+^ at 646.4 eV. For Mn2p3/2, it can be deconvolved into two peaks at 640.6 eV and 642.1 eV, corresponding to Mn^2+^ and Mn^3+^, respectively; for Mn2p1/2, it was attributed to Mn^2+^ and Mn^3+^ when the binding energies were 652.2 eV and 653.8 eV. The C1s in the biochar was divided into three peaks. FBC at the binding energies of 285.08 eV, 286.88 eV, and 289.28 eV corresponded to the characteristic peaks of C-C/C=C (graphite carbon), characteristic peaks of C-O (alcohol, phenol, or ether group), and O-C=O (metal carbonate or carboxylic group), respectively [20,21,22]. The C-C content in KFBC decreased from 84.21% to 74.08%. Although the content of C=O decreased and the content of C-O increased, the overall proportion was higher than that of the unmodified magnetic biochar, indicating that the surface of the bimetal-loaded biochar increased, just as in the characterization of the IR analysis. FBC corresponded to C-OH (surface hydroxy oxygen) at 533.43 eV, C=O (chem O) at 533.43 eV, and M-O (lattice oxygen) at 533.43 eV. The lattice oxygen ratio in the KFBC was increased from 7.76% to 18.78%, due to the more metal oxides present on the biochar surface. The proportion of the surface hydroxyl groups on the KFBC was increased, probably because of the strong oxidative nature of the KMnO_4_, increasing the oxygen-containing functional groups on the surface. The fine spectrum of Fe2p is shown in Figure 6: Fe2p3/2 at the binding energy of 711.3 eV, a satellite peak at 718.8 eV, and Fe2p1/2 at 724.9 eV. Peaks at 711.13 eV corresponded to Fe^2+^, and peaks at 714.2 eV corresponded to Fe^3+^. For Fe2p1/2, the peaks at 724.23 eV and 727.38 eV were attributed to Fe^2+^ and Fe^3+^, respectively. The proportion of Fe^3+^ in the modified magnetic biochar was increased, and the proportion of Fe^2+^ was decreased, indicating that the average valence of Fe was increased. 

**Figure 7:**
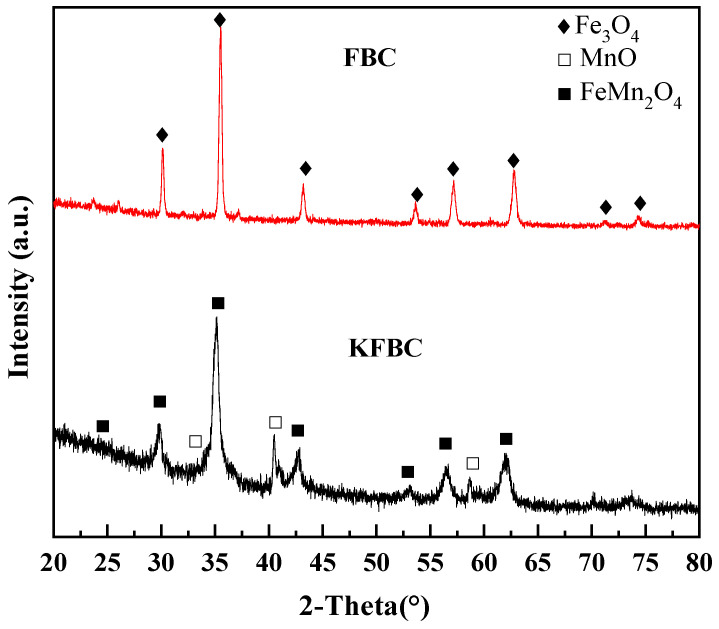
XRD spectra of magnetic biochar.

The XRD spectrum of the biochar is shown in Figure 7, where the characteristic peaks of amorphous charcoal appear, indicating that the biochar obtained by pyrolysis was amorphous, corresponding to the Raman spectrum. The presence of many strong diffraction peaks in the spectrum indicated the presence of mineral crystals in the material. The successful attachment of metal oxides to the surface of the biochar occurred after modified pyrolysis. The additional loading of MnO and FeMn_2_O_4_ on the surface of KFBC compared to FBC proved the successful loading of iron manganese oxides on the surface of the biochar [23].

### 2.2. Pb^2+^ Adsorption Tests

The pH value can affect the surface charge density of the adsorbent and the present form of Pb^2+^. The variation of the adsorption capacity of the biochar at different pH values is shown in Table 2. At pH = 2, both FBC and KFBC exhibited lower adsorption capacity, with a decrease of 14.671% and 31.410%, respectively, compared to the maximum adsorption capacity, indicating that the modified biochar was greatly affected by the change of pH value. On the one hand, this is because the surface of the biochar was positively charged and it was difficult for the surface oxygen-containing functional groups to ionize hydrogen ions, thus hindering the complexation of Pb^2+^ with oxygen-containing functional groups. On the other hand, the adsorption effect of the modified biochar was strongly influenced by pH, probably because the metal oxides, which play a dominant role in the adsorption process, were corroded in solutions with lower pH and dissolved into the solution as free ions, resulting in lower adsorption. The best adsorption of Pb^2+^ was achieved at pH = 5, when the adsorption amounts of FBC and KFBC were 27.373 mg/g and 113.172 mg/g, respectively. Some studies showed that at pH > 5.8, Pb^2+^ began to precipitate and attach to the surface of the biochar, blocking the pore channels and inhibiting the diffusion of lead ions, and at pH = 6, the adsorption amount of Pb^2+^ by the biochar was, instead, decreased. 

To investigate the effect of different species and different strength ion concentrations on the adsorption of Pb^2+^, different molar concentrations of K^+^, Na^+,^ and Ca^2+^ were mixed with Pb^2+^ solutions; the changes in the adsorption amount are shown in Table 3. The results showed that low concentrations of K^+^ and Na^+^ had less effect on the adsorption of Pb^2+^ due to their lower charge density, which did not compete with the adsorption of Pb^2+^. On the other hand, the forces between K^+^ and Na^+^ and the water molecules were stronger than those with the adsorbent; so, the effect on the adsorption of Pb^2+^ was not significant. However, when the concentration was high, the adsorption of Pb^2+^ by the biochar decreased slightly, probably due to the formation of off-sphere complexes. The influencing ions in the solution occupied the adsorption sites in the outer layer of the biochar, competing with the adsorption of the heavy metal ions.

The presence of Ca^2+^ can have an effect on Pb^2+^. It has been shown that the presence of Ca^2+^ affects the removal of pollutants by adsorbents [24,25]. At low Ca^2+^ concentrations, the effect on the adsorption of Pb^2+^ is not significant. However, at its concentration comparable to that of Pb^2+^, the adsorption of Pb^2+^ decreases significantly. This phenomenon can be attributed to the fact that high concentrations of Ca^2+^ will compete with Pb^2+^ for adsorption sites, Ca^2+^ will affect the activity coefficient of Pb^2+,^ and Ca^2+^ will limit the precipitation of Pb^2+^ on the adsorbent surface. Meanwhile, Ca^2+^ in water can have an impact on the aggregation behavior of inorganic nanoparticles.

### 2.3. Adsorption Kinetics

**Figure 8:**
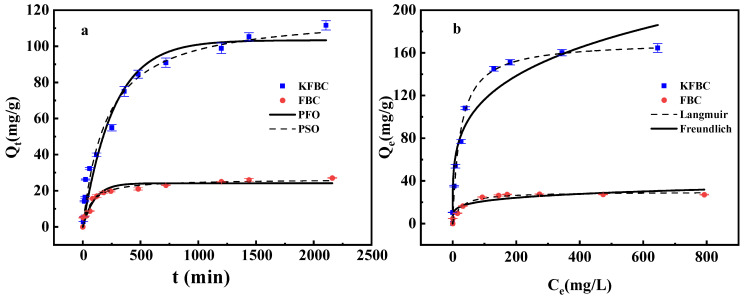
Adsorption kinetics of FBC and KFBC (**a**), adsorption isotherms of FBC and KFBC (**b**).

The PFO model assumes that the diffusion rate of pollutants is the main factor affecting the adsorption rate. The PSO model (PSO) is mainly controlled by the complexation or ion exchange between adsorbate and adsorbent. The fitted curves for the PFO and PSO of the heavy metal adsorption process on the biochar are shown in Figure 8, and the fitted parameters are shown in Table 4. The fitted correlation coefficients for the PSO kinetics (R^2^ = 0.964, 0.979) were both greater than the PFO kinetics (R^2^ = 0.959, 0.933). It indicates that the adsorption rate of the biochar was mainly controlled by chemisorption and that the modification did not change the adsorption mode, with a slight decrease in the adsorption rate of KFBC.

### 2.4. Adsorption Isotherms

As can be seen from Figure 8, the adsorption increased rapidly with an increasing equilibrium concentration at lower Pb^2+^ concentrations; at higher concentrations, the adsorption increased less. As shown in Table 4, the model fit revealed that the Langmuir model was a better fit than the Freundlich model for both adsorbents, indicating that the process was dominated by monolayer adsorption with a maximum theoretical adsorption capacity of 170.668 mg/g. The K_L_ of both FBC and KFBC was less than 1, indicating that both adsorbents could effectively adsorb Pb^2+^. The K_L_ of KFBC was larger than that of FBC, indicating that KFBC was more favorable for the adsorption of Pb^2+^, which was consistent with the actual experimental results.

### 2.5. Adsorption Mechanism of Pb^2+^

**Figure 9:**
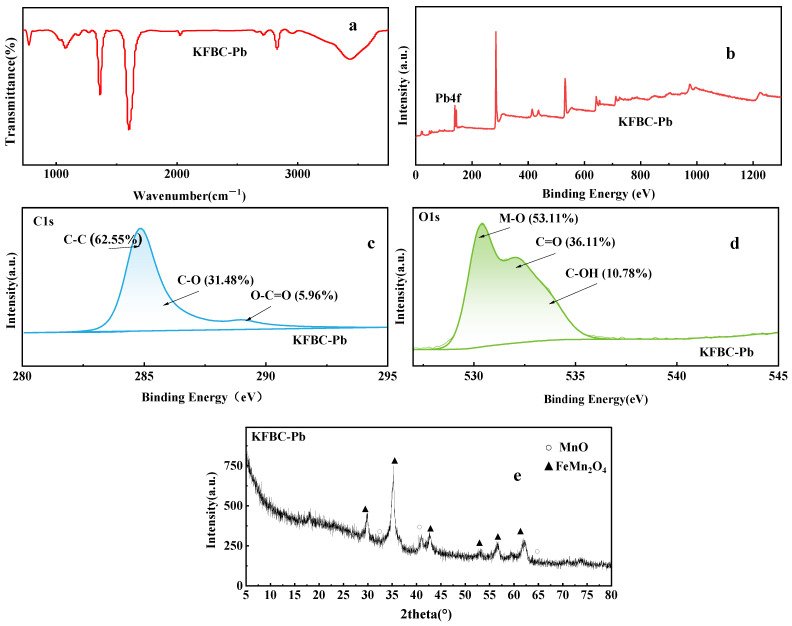
Comparative characterization of KFBC after adsorption by (**a**) FTIR, (**b**) XPS, (**c**) C1s of XPS, (**d**) O1s of XPS, (**e**) XRD.

The increase in adsorption was mainly due to the increased functional groups on the surface of the biochar after modification with KMnO_4_ and the loading of manganese oxides. The changes of the oxygen-containing functional groups on the surface of the biochar after adsorption are shown in Figure 9a. The blunting of the functional groups after adsorption and the weakening of the intensity of the hydroxyl and carboxyl peaks indicated that the adsorption depleted the functional groups on the surface of the biochar. In combination with XRD (Figure 9e), no new peaks appeared on the KFBC surface, indicating that precipitation played a minor role in the adsorption of Pb^2+^ on KFBC.

The fine spectra of C1s after adsorption are shown in Figure 9c. The fitted results of C1s showed that the characteristic peaks of both C-O and C=O of the biochar were displaced and the peak intensities changed after adsorption, which may have been due to the involvement of hydroxyl and carboxyl groups on the surface of the biochar in the reaction. The fine spectra of O1s after adsorption are shown in Figure 9d. Compared to the pre-adsorption period, the characteristic peak areas of lattice oxygen all more than doubled, indicating the formation of Pb-O on the surface of the biochar. The area of the C=O peak also increased, suggesting both monodentate and bidentate chelation on the surface of the biochar, while the characteristic peak areas of C-OH all decreased. Presumably, -OH was involved in the adsorption process of the biochar, which corresponded to the results of the IR spectra.

**Figure 10:**
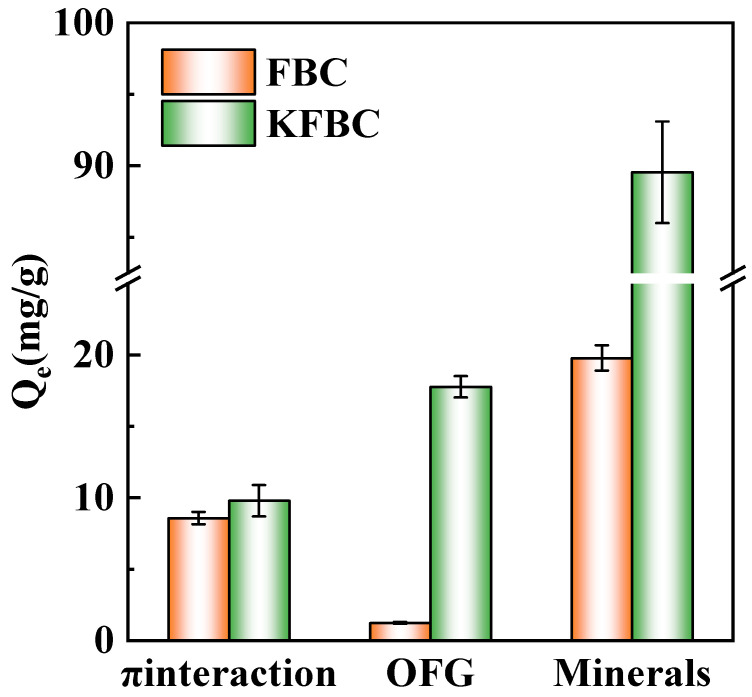
Quantitative study of Pb^2+^ adsorption mechanism on FBC and KFBC compared with other adsorbents.

The adsorption of the biochar consisted of π-electron interactions, oxygen-containing functional groups, and inorganic minerals; the amount of adsorption dominated by each component is shown in Figure 10. The π-electron interactions dominated the adsorption with little difference, and the aromatic structure was related to the pyrolysis temperature of the biochar, which can act as a π-electron donor [26]. Additionally, it was shown that π-electron-dominated adsorption was related to the pyrolysis temperature [27]. The increase in the oxygen-containing functional groups of the modified biochar resulted in an increase in the oxygen-containing functional group-dominated adsorption of Pb^2+^ from 1.242 mg/g to 17.77 mg/g. The inorganic mineral-dominated adsorption was stronger, and the ash-dominated adsorption of KFBC increased by more than 3.5 times compared to that of FBC. 

The adsorption capacity of other biochar adsorbents reported in the literature for Pb was compared. In general, the maximum adsorption capacity of KFBC was acceptable. The structural characteristics of the various adsorbents and their adsorption capacities under optimal adsorption conditions are shown in Table 5. Compared with other adsorbents, KFBC may not be the best, but its main advantage is that it can be obtained in large quantities as a raw material and can also be recycled and reused, while reducing environmental pollution.

## 3. Materials and Methods

### 3.1. Preparation of Biochar

According to the previous preparation method and making some modifications [31], coconut peel was washed and dried in an oven at 105 °C for 24 h, after which it was crushed through a 20-mesh sieve to produce a reddish-brown coconut peel powder. Then, it was sealed and placed in a desiccator for later. The coconut peel was placed in a corundum boat and pyrolyzed at 600 °C for 2 h under N_2_ atmosphere, yielding a 37.06% coconut peel biochar. According to the mass ratio of biochar to Fe(NO_3_)_3_·9H_2_O 1:1, 5.61 g of coconut peel powder was mixed with 2.02 g of Fe(NO_3_)_3_·9H_2_O in an ethanol solution and stirred magnetically at room temperature for 24 h. The suspension was ultrasonically dispersed for 1 h and dried to a constant weight in an oven at 65 °C. The dried mixture was placed in a corundum boat and then placed in a quartz tube furnace under N_2_ atmosphere at a heating rate of 10 °C/min to 600 °C and pyrolyzed for 2 h. The magnetic biochar obtained after carbonization was ground, passed through a sieve of 200 mesh, rinsed with deionized water until the surface pH was not changing, dried to constant weight in an oven at 105 °C, and labeled as FBC. The carbon-modified biochar was prepared in the same way as FBC, except that 0.79 g of KMnO_4_ was added during the impregnation process according to the molar ratio of Fe(NO_3_)_3_·9H_2_O to KMnO_4._

### 3.2. Adsorbent Characterization

To analyze the morphology and properties of the adsorbent, the biochar sample needed to be characterized. The BET surface area, pore size distribution, and total pore volume were characterized by performing N_2_ adsorption–desorption (IQ-2). Scanning electron microscopy (SEM) (S-4800) was used for the surface morphology, and elemental composition analysis was measured by X-ray photoelectron spectroscopy (XPS) (ESCALAB 250Xi). The chemical functional groups of FBC and KFBC were ascertained by FTIR spectra (ALPHA-T, Bruker Instruments, Germany). The concentration of Pb^2+^ was measured using an inductively coupled plasma emission spectrometer (Optima 8000). The standard curve should be prepared before the test. The Pb^2+^ standard solution (1 g/L) was diluted to 0, 1, 3, 5, and 10 mg/L, and the Pb^2+^ concentration was determined. To reduce errors, three parallel samples were set up for each group of tests.

### 3.3. Adsorption Experiments

#### 3.3.1. Configuration of Pb^2+^ Stock Solution

An appropriate amount of deionized water was used to dissolve 1.598 g Pb(NO_3_)_2_ and was diluted into 1 L volumetric flask with 1 M HNO_3_ (pH = 5). The concentration of Pb^2+^ stock solution was 1 g/L, and the concentration of Pb^2+^ solution required for subsequent experiments was obtained by the dilution of this stock solution.

#### 3.3.2. Effect of Different Solutions’ pH on the Adsorption Effect

The initial concentration of Pb^2+^ solution was adjusted to about 200 mg/L. The pH of the Pb^2+^ solution was adjusted to the desired values (2, 3, 4, 5, 6) with a 1M HCl and NaOH solution, and 10 mg of FBC and KFBC were added to different pH values of Pb^2+^ solution. A blank experiment without the addition of biochar was carried out at the same time; the samples were placed in a water bath shaker (25 °C) with a plug and shaken at 200 r/min for 36 h. The heavy metal adsorption capacity of the biochar is calculated as in Equation (1):

(1) $$q_{e}=\frac{\left(C_{0}-C_{e}\right)\;V}{M}$$

where q_e_ (mg/g) is the adsorption capacity at equilibrium of the biochar on Pb^2+^, C_0_ (mg/L) is the initial concentration of Pb^2+^, C_e_ (mg/L) is the concentration of the Pb^2+^ solution at adsorption equilibrium, V (mL) is the volume of the Pb^2+^ solution, and m (mg) is the quality of biochar added.

#### 3.3.3. Adsorption Kinetics

The adsorption kinetics can directly reflect the adsorption mechanism, which is the main factor related to the physical and chemical properties of the adsorbent. A solution with a Pb^2+^ concentration of 200 mg/L was prepared. The initial pH of the solution was adjusted to 5 with 1 M HNO_3_. Then, 300 mg of FBC and KFBC were taken with 300 mL of the solution in a conical flask, and samples were taken at predetermined time intervals (0.5–48 h). The samples were passed through 0.45 μm filter membranes for solid–liquid separation to determine the Pb^2+^ concentration. The adsorption kinetic curves were fitted using the pseudo-first-order (PFO) kinetics model and the pseudo-second-order (PSO) kinetics model. The specific equation is as follows:

(2) $$\mathrm{PFO}:\;q_{t}=q_{e}\left(1-e^{-k_{1}t}\right)$$

(3) $$\mathrm{PSO}:\;q_{t}=\frac{k_{2}q_{e}^{2}t}{1+k_{2}q_{e}t}$$

where k_1_ (min^−1^) and k_2_ (g/(mg·min)) represent the reaction rate constants of the first-order and second-order kinetic equations, t (min) is the reaction time, and q_t_ and q_e_ (mg/g) are the adsorption capacities at time and the completion of adsorption equilibrium, respectively.

#### 3.3.4. Adsorption Isotherm

Adsorption isotherm is a model that describes the adsorption capacity per unit mass of the adsorbent in relation to the concentration of pollutants in the solution at a constant temperature; it can reflect the adsorption capacity of the biochar and the interaction between heavy metals and biochar. Pb^2+^ solutions with concentrations ranging from 0 to 800 mg/L were prepared, and the pH of each group was adjusted to 5. Then, 10 mL of Pb^2+^ solutions with different concentrations were added to a 15 mL centrifuge tube; 10 mg of FBC and KFBC were added, respectively, and shaken at room temperature for 24 h. The adsorption isotherms were fitted using the Langmuir and Freundlich models. The specific equation is as follows:

(4) $$\mathrm{Langmuir}\;\mathrm{model}:\;q_{e}=\frac{Q_{0}K_{L}C_{e}}{1+K_{L}C_{e}}$$

(5) $$\mathrm{Freundlich}\;\mathrm{model}:\;q_{e}=K_{F}C_{e}^{1/n}$$

where C_e_ (mg/L) is the concentration of the adsorbate at saturation, K_F_ (mg/g) is the adsorption capacity, n is a constant, Q_0_ (mg/g) is the maximum adsorption capacity of Langmuir, and K_L_ (L/mg) is the Langmuir constant.

#### 3.3.5. Effect of Coexisting Cations on Adsorption

In order to determine the effect of coexisting ions on the removal of Pb^2+^, K^+^, Na^+,^ and Ca^2+^ were mixed into the Pb^2+^ solution with an initial concentration of 200 mg/L and pH of 5; the concentration of coexisting ions was 0, 0.2, 1, and 5 mM [32]. Then, 10 mL of the mixed solutions were added to 15 mL centrifuge tubes; 10 mg of FBC and KFBC were added, respectively. The mixtures were plugged into a water bath shaker (25 °C) and shaken at 200 r/min for 36 h. The concentration of Pb^2+^ was measured after filtration.

### 3.4. Quantitative Analysis of Different Mechanisms to Pb^2+^

Different mechanisms contribute to Pb^2+^ adsorption, and the calculation of the contribution of different mechanisms to Pb^2+^ on the biochar was reported [33]. The adsorption mechanism was classified into three parts: adsorption attributed to the minerals including MnO_x_ (Q_m_), oxygen functional group complications (Q_f_), and coordination of heavy metals with π electrons’ (Pb^2+^-π) interaction (Q_p_). FBC and KFBC were mixed with 1 M HCl at a solid–liquid ratio of 1:15 and shaken for 24 h in a shaker. Demineralization was carried out, and the functional groups on the surface of the biochar were not changed at this time. The reduced adsorption amount after demineralization was the heavy metal adsorption amount dominated by mineral precipitation. The amount of heavy metal adsorbed after demineralization was the amount of heavy metal adsorbed by mineral precipitation. The mechanism of heavy metal adsorption after mineral precipitation removal included the complexation of the oxygenated functional groups and π-electron coordination. In the process of oxygen-containing functional group complexation, hydroxyl and carboxyl groups release H^+^ into the solution through exchange. Therefore, the decrease in pH of the solution before and after adsorption was measured by demineralized biochar. The decrease in pH of the solution before and after adsorption was measured to calculate the amount of H^+^ released; accordingly, the amount of Pb^2+^ and the remaining part was considered as π-electron coordination adsorption [34]. Concisely, the contribution of the minerals (Q_m_) was calculated by the difference of the adsorption capacity of the adsorbent (Q_t_) and the acid washing adsorbent (Q_a_). The contribution of the oxygen-containing functional groups (Q_f_) was calculated by the released H^+,^ which is caused by the process of complex oxygen-containing functional groups during the de-ashed biochar adsorption process. Finally, the π interaction part (Q_p_) was the difference of the Q_a_ and Q_f_. The specific quantitative analysis is considered as follows:

Q_m_ = Q_t_ − Q_a_ (6)

Q_p_ = Q_a_ − Q_f_ (7)

### 3.5. Data Analysis

The experiment was carried out three times and the experimental data were expressed as “mean ± standard error”. The data were organized using Excel 2013 software. XRD data were analyzed using Jade 6.5 contrast cards. The XPS was fitted to the split peaks using Avantage. The analyzed data were plotted using Origin 9.0 software. 

## 4. Conclusions

In this study, organic solid waste was thermochemically treated for high-value utilization. KFBC, the KMnO_4_-modified magnetic biochar, was successfully synthesized for Pb^2+^ adsorption. The pseudo-second-order kinetic model and the Langmuir isothermal model fitted the Pb^2+^ adsorption process well. The adsorption process on the surface of KFBC was homogeneous adsorption, and chemical adsorption dominated the adsorption process. FBC and KFBC had the best adsorption effect on Pb^2+^ at pH = 5. High concentrations of k^+^, Na^+^, and Ca^2+^ metal ions competed with Pb^2+^ for adsorption, resulting in lower adsorption capacity. The adsorption inhibition effect of Ca^2+^ was relatively obvious. The complexes with MnOx and oxygen-containing functional groups enhanced the adsorption process, and the analysis of the adsorption mechanism showed that electrostatic interaction also played an important part in removing Pb^2+^.The modified magnetic biochar had a good adsorption performance for Pb^2+^ in wastewater. Subsequently, a variety of heavy metals and organic substances should be added to investigate the selective adsorption capacity of magnetic biochar for Pb^2+^ in the coexistence of other heavy metals or organic substances, so as to provide a basis for the application of magnetic biochar in engineering. This will provide a basis for the application of magnetic biochar in engineering.

## Figures and Tables

**Table 1:** Analysis of surface area and pore size of different biochars.

	S_BET_ (m^2^/g)	V_tot_ (cm^3^/g)	Pore Width (nm)
FBC	234.350 ± 10.745	0.030 ± 0.001	4.990 ± 0.3354
KFBC	246.320 ± 13.331	0.053 ± 0.002	5.170 ± 0.2716

The data represent the mean ± standard deviation of three replicates.

**Table 2:** Influence of solution pH on Pb^2+^ removal by FBC and KFBC.

	pH	Adsorption Amount of Pb^2+^(mg/g)
FBC	2	23.357 ± 1.034
	3	26.375 ± 1.958
	4	26.968 ± 2.444
	5	27.373 ± 2.954
	6	26.425 ± 1.002
KFBC	2	77.625 ± 2.382
	3	98.958 ± 3.985
	4	108.2875 ± 4.514
	5	113.172 ± 4.526
	6	84.183 ± 4.091

The data represent the mean ± standard deviation of three replicates.

**Table 3:** Effect of different molar concentrations of alkali metal ions on the adsorption of Pb^2+^ by KFBC.

	Ion Concentration(mM)	Adsorption Capacity of Pb^2+^(mg/g) by KFBC
K^+^	0	111.521 ± 5.659
	0.2	107.531 ± 4.763
	1	106.333 ± 4.368
	5	105.532 ± 1.562
Na^+^	0	111.578 ± 5.341
	0.2	110.759 ± 5.267
	1	108.434 ± 5.031
	5	107.397 ± 3.953
Ca^2+^	0	111.534 ± 5.555
	0.2	105.204 ± 4.005
	1	98.828 ± 2.597
	5	90.858 ± 2.658

The data represent the mean ± standard deviation of three replicates.

**Table 4:** Regression parameters of Pb^2+^ adsorption kinetics and Pb^2+^ adsorption isotherm.

**Pb^2+^**	**PFO**			**PSO**		
	q_e_	K_1_	R^2^	q_e_	K_2_	R^2^
FBC	24.159	0.0097	0.959	26.402	0.0005	0.964
KFBC	103.334	0.0038	0.933	118.188	0.0004	0.979
Pb^2+^	Langmuir			Freundlich		
	Q_0_	K_L_	R^2^	K_F_	n	R^2^
FBC	29.606	0.0408	0.975	36.271	3.958	0.945
KFBC	170.668	0.0409	0.990	8.406	5.013	0.897

**Table 5:** Adsorption capacity of various adsorbents reported in the literature.

Adsorbent	Adsorbent Raw Materials	Surface Area (m^2^/g)	q_e_ (mg/g)	References
KFBC	Coconut peel	246.320	170.668	This paper
FW700C	Waste fir wood	343	39.31	(Dong et al., 2022) [28]
MWCNTs@SiO_2_-NH_2_	Carbon nanotubes	86.3	147	(Yang et al., 2018) [29]
AABC	Activated biochar	75.92	98.33	(Wang et al., 2018) [20]
Fe_3_O_4_/Cu-MOFs	Magnetic metal organic frameworks	35.4	219	(Shi et al., 2018) [30]

## Data Availability

Not applicable.
